# The Dynamics of Visual Art Dialogues: Experiences to Be Used in Hospital Settings with Visual Art Enrichment

**DOI:** 10.1155/2011/204594

**Published:** 2011-12-22

**Authors:** Britt-Maj Wikström

**Affiliations:** ^1^Department of Health, Nutrition and Management, Faculty of Health Sciences, Oslo and Akershus University College of Applied Sciences, 0130 Oslo, Norway; ^2^Department of Public Health Sciences, Karolinska Institute, 17177 Stockholm, Sweden

## Abstract

*Objectives*. Given that hospitals have environmental enrichment with paintings and visual art arrangement, it would be meaningful to develop and document how hospital art could be used by health professionals. *Methods*. The study was undertaken at an art site in Sweden. During 1-hour sessions, participants (*n* = 20) get together in an art gallery every second week five times. *Results*. According to the participants a new value was perceived. From qualitative analyses, three themes appear: raise association, mentally present, and door-opener. In addition 72% of the participants reported makes me happy and gives energy and inspiration, and 52% reported that dialogues increase inspiration, make you involved, and stimulate curiosity. *Conclusion*. The present study supported the view that visual art dialogue could be used by health care professionals in a structured manner and that meaningful art stimulation, related to a person's experiences, could be of importance for the patients. Implementing art dialogues in hospital settings could be a fruitful working tool for nurses, a complementary manner of patient communication.

## 1. Introduction

Many hospitals have environmental enrichment with paintings and visual art arrangement that can be used by health care professionals in a structured manner. One way to use hospital art could be that health professionals start a dialogue with a patient inspired by a painting. The role of health care professionals could be to support and encourage the patient to combine earlier memories and experiences with new impressions from the painting.

The present line of research may prove fruitful in that it could inspire health professionals to seek new directions by which art could be integrated in nursing care. Many hospitals have environmental enrichment with paintings and visual art arrangement that can be used by health care professionals in a structured manner in order to develop nursing care. One way could be to use hospital art to start a dialogue inspired by a painting.

The relevance of art in nursing care needs to be considered, particularly because it provides a lens through which an alternative nursing care could improve patient care. Health professionals have to take into consideration that patients do need different ways to express their feelings. Communication via a work of art could be one way to communicate with patients. It could be alternative non-stigmatizing environments for helping people with mental health problems understand emotional distress. Health professionals could ask the patient to “think aloud” what they see, think, and feel about the artworks that might expand the patient's thoughts and, therefore, give health care professionals strategies they need to understand the patient. The development of this understanding is theoretically based and a value of significance in developing nursing care. For the patient, being able to both feel and talk about the characteristic of their life situation could be a help in nursing care. A beneficial effect of art communication is to be found in research of today [1].

## 2. Background

The research project has designed an approach to scientifically evaluate the effect of visual art dialogues performed over a period of time. This should be a subject of concern for a wide range of professionals including nurses, policy makers, artists, and administrators.

The present study explores visitor's perception when a structured art dialogue (SAD) model was used. The integration of the SAD model will provide an ideal setting for answering the question of the value of the SAD model. The works of art used in the present study are by a Swedish painter, Anna Toresdotter.

The European Charter on Environment and Health declares that good health and well-being require a harmonious environment in which aesthetic, social, physical, and psychological factors are important. The integration of the SAD model can play a crucial role in achieving these objectives. Evidence is needed to demonstrate that the integration of art dialogues could include beneficial effects for the spectator's perception of psychological and physiological health. Several studies show that art dialogues, with patients living in a block of specially designed flats, proved to be a way to stimulate communication [1–5].

The meaning of aesthetics has been formulated by ancient philosophers such as Aristoteles [6] and is to be found in more modern research [7–15]. They saw a natural link between art and life. Painting, drama, dance, and music were obvious parts of everyday life and were regarded as healing for the body and mind. Art is timeless, and artistic expression has been in existence throughout mankind's history. It has aesthetic value, and its crucial purpose is to provide aesthetic experience and to encourage creativity in the viewer.

Studies about how to use works of art has shown positive learning effects in nurse education. The studies are based on the thinking that the way the students see things is affected by what they know, pointing at the relation between what the paintings represent and the students' knowledge. The painter's way of seeing is presented in a painting, and the students' perception or appreciation of the painting depends upon the painter's way of seeing as well as the students' experiences.

Decoding nonverbal communication via works of art was a valuable tool additional to theoretical knowledge in order to prepare student nurses for their profession [16, 17]. Edward Munch's painting “The Sick Girl” stimulated student nurses to engage in learning about empathy. A control group was used to control for the effects of the visual art dialogues. Students in the visual art group were more engaged in learning about empathy [18]. Art dialogues at a museum were used in order to learn about interpersonal relations. The result based on three steps, observation, conceptualisation, and reflection, shows that student nurses became aware of interpersonal relations in works of art that communicate a broad spectrum of human experiences and thoughts [19–22]. Works of art proved to increase learning outcomes when added to theoretical knowledge when teaching nursing care [23–26].

Art is important in the empowerment process [27], and effects are found on leaders and coworkers in an art-based leadership development program [28]. Art is of importance for health and psychological well-being. Similar finding are reported about environments that are healing [29, 30].

The meaning of aesthetics for physical and psychological health as it has been described by ancient philosophers is to be found in the research of today. For instance, aesthetic forms of expression for elderly individuals can mean discovering, preserving, or developing possibilities for a meaningful life. Several controlled intervention studies [19, 31] describe beneficial effects in elderly individuals' health. Dialogues generated by reproductions of works of well-known artists had a positive impact on elderly individuals' perceptions of their life situation and social interaction compared to a control group in which dialogues were about events of the day and the elderly individuals' hobbies and interests. In a survey of living conditions [32], 12,675 people were interviewed, and a followup was conducted concerning survival. The results showed that attendance at cultural events, which included visiting museums, theatres or concerts, reading books, and singing in a choir, had a positive influence on survival rates.

Dewey dealt with human experience [11]. He viewed aesthetic experiences and peak experiences, as particular kinds of experience, and an interaction between a human being and some aspects of the environment. Reflecting on an experience after its occurrence according to Dewey is neither emotional nor intellectual alone; it is an intensified form of an ordinary experience that belongs not only to the museums, but also to general experiences that culminate in aesthetic experience. He also spoke of aesthetic peak experiences as moments of joy and temporary loss of time and self-awareness. For instance, individuals could feel a day passing as if it were only a few minutes. In such moments, reality is perceived as good and desirable, and the experience could be so valuable as to make life valuable. There are many beneficial after effects of aesthetic peak experiences: individuals' views of themselves, others, and the world that might be changed in a healthy direction [11].

The investigation in the present study was aimed toward constructing an SAD program. The program included a verbal-visual procedure, characterized by dialogues based on the impressions generated from paintings, experienced-based and allowed spectators to express issues of individual value.

There is a need to further develop knowledge from previous research about visual art communication. Looking at a work of art and into the reality of other times, places, and people is to connect the past and the present and to integrate pieces of experience into a whole.

The question to be answered in the present study is whether the SAD program has positive communication aspects.

## 3. Material and Methods

The present study was undertaken at an art gallery in Sweden. The population studied was men and women with no formal education in art but interested in art and visiting art galleries. During a 1-hour session, participants meet in a visual art gallery every second week five times. The group was exposed to guided dialogues generated from the impression of the works of art. The gallery was situated outside a city and has a view over water, hills, and flowers.

### 3.1. Recruiting Participants

Method used to collect data was accidental sampling, a sample of convenience, the most readily available persons. In the present study, it meant that participants were recruited via two galleries from their mailing lists and from persons who worked at a public department. Participants who were interested to participate were invited to a meeting to get information of the study. At this meeting the project leader and organizers presented themselves, the aim, and procedure of the project. The information was also given to each participant in written form.

### 3.2. Structure of Data Collection

Participants were requested to describe, in their own words, the meaning of looking at and discussing paintings at a visual art gallery.

Data was collected by questions to be answered by participants during the art intervention period:

the meaning of looking at and discussing paintings in structured form at a visual art gallery,
The meaning of music, reading, movie, and theater in life,
the meaning of gallery visits with structured dialogues compared to gallery visits without structured dialogues,

Two month after the last art intervention meeting participants answered the following questions.

 Which art form do you prefer?

 How should this art form be developed in order to reach many people? 

Why is the art form you prefer important for you?

The art form you prefer—has your preferences changed during the art dialogues?

In addition, a copy of a painting was mailed to each participant. A new copy was sent in between the gallery visits that took place every second week (*n* = 5). Participants were asked to write down what comes to their mind when looking at the copy and send their reflections to the project leader.

### 3.3. Analysis of Data

Data was analyzed in three steps: create a first impression of the interview, find the underlying message in the statements representing different dimensions, and formulate categories by reading text repeatedly to get an understanding behind the words.

The starting point for the analysis is qualitative, which means that the researcher is open to the text and does not make use of any theory or preconceived ideas in order to understand the text. In this study, the term theory means any explicitly defined hypothetical construction.

A qualitative analysis of the semistructured feedback form was performed in several steps using the analytical technique [33–35]. A close examination of the data was conducted and compared for both similarities and differences. The first step was a content analysis.

Each of the written accounts was read several times in order to grasp the content. Secondly, each account was studied to qualitatively identify different comments which together comprise the total account. Each comment was considered as a unit of analysis and defined as an utterance that provided new information about the participants' impressions and opinions [36]. Questions were asked about the phenomena as reflected in the data and reviewed for emerging themes and were categorized, coded, and counted. The open coding classified the data and allowed for the identification of categories. Even so, the researcher's cultural and linguistic understanding of the phenomenon was the prerequisite for coming to an understanding of the participants' accounts.

### 3.4. Reliability and Validity

There can be no validity without reliability, thus demonstrating that validity is sufficient to establish reliability [37]. The term dependability is used in qualitative research, which closely corresponds to the notion of reliability in quantitative research.

In the present study this aspect is considered to mean that reliability is a consequence of validity. In the present study, the criteria for establishing trustworthiness are credibility and dependability. Credibility is ensured by accurately describing and identifying those participating, with dependability relying on credibility. A qualitative research study that establishes credibility will also be dependable [37]. In the present study, dependability was assured by following a clear research procedure and discussing decisions taken about theoretical choices with a research colleague.

The analyses were used to ensure that the meaning units in a category were not only derived from one semistructured formula. By doing this, it was possible to obtain consistency from the categories. Although the categories were consistent and valid, all the aspects of the material were not clearly displayed since the aim of describing the meaning of the SAD model is a complex procedure.

### 3.5. Intervention Procedure

When the participants met, they listen to music for five minutes. Then they look at the exhibition together with the artist. They walk around and then chose a painting that captures their interest. It is the participants alone who decide which of the paintings they choose to look at and to talk about. The art dialogues, generated from paintings, focus on the spectators own resources and what they see in the painting.

A new exhibition was shown at each of the five meetings. The exhibition constitutes of paintings, dance suits, and sculptures in papier-mâché. The artist introduces a video showing a dance performance. This lasted for five to ten minutes. Then the participants choose a painting that captures their interest. If they wanted, they could be inspired by following request: describe the visual art object, what it represents, colour and forms! Pretend you are the artist and know all about it, what does it brings to your mind, do you want to change something in the painting! The artist gave a description of her thoughts when participants asked her, but pointed out that there is no explicit meaning of the painting. This model is based on previous research [3, 18, 24].

Between the five meetings, three times, participants were asked to reflect over a painting sent as a mail and respond to the project leader via mail.

### 3.6. Description of the Paintings: Degree of Complexity in the Chosen Paintings

The art works are lithography and oil paintings. Some paintings were representative, with different degree of representatively. The motifs were flowers and human beings. Some of the paintings were abstract.

The choice of paintings was be based on the knowledge that patterns are judged interesting when they contain information that cannot be absorbed immediately but seem likely to be absorbed relatively quickly through perceptual and intellectual efforts. Complexity, ambiguity, and variability are associated with a high degree of uncertainty, novelty, and surprise with high information content. It is important that the information content and the uncertainty in a visual art object is neither too low or too high but in balance with a person's ability to perceive it. Therefore, the selection of visual art objects must be made with care, in collaboration with the person who looks at it [38–40].

The rules were followed regarding the participants receiving the usual assurance about anonymity, confidentiality, and the right to withdraw at any point without prejudice.

Comments are directly quoted, while always ensuring that the speaker is not identified.

From an ethical perspective, the qualities as judged by the Declaration of Helsinki [41] are that the research design and the need in society for such a project are deemed to be of importance.

An ethical issue that could arise from participants in the present study was that participants were in a state of dependence in relation to the leader who was in charge of the gallery as well as has made the paintings. However, participants were not asked to give opinions about the paintings aesthetical or formal value. In addition, the collected data was sent to the leader researcher anonymously.

The purpose and procedure were carefully explained to the participants, who were assured of confidentiality. An ethical issue that could arise from the participants was that they were in a state of dependence in relation to the leader in charge of the gallery, particularly with concern to a question in which the participants were requested to describe the meaning of the structured art dialogues.

## 4. Results

The results constitute of four pats based on questions to be answered by participants in written form.

1. The meaning of looking at and discussing paintings in structured form at a visual art gallery.
2. The meaning of music, reading, movie, and theater in life.
3. The meaning of structured dialogues compared to a nonstructured dialogues.
4. Reflections two months after the study were finished; which art form do you prefer? How should this art form be developed in order to reach many people? Why is the art form you prefer important for you? The art form you prefer—has your preferences changed during the art dialogues?

### 4.1. Part 1

**Based on Question 1**

Participants were requested to describe, in their own words, the meaning of looking at and discussing paintings. The results are based on citations from verbatim expressions. The results are presented in three subthemes.

**Raise Association**

… the coffee cup … classical design … makes me remember ….
… it is … really nice to discover details … of old furniture … from early days.
… the painting … show the precision … of people … forgotten … long ago ….
… the painting … makes me remember … I have never thought of … since I was four or five years of age.
… it makes me remember a stable … an accident … riding … about horses.
… when I saw … painting … fishing with …, associated to hitchhiking … when very young.

**Door-Openers**

… in order to have some help … makes the conversation much easier.
… I like this way to get close to the painting … is a way to my inner life.
… it is easy to open a conversation … leads to further conversation … one subject leads to another subject.
… I assume … I am not satisfied … want to know … what means the trancelike figure … it is something to discuss.
… I like some help … the painting is a link to my inner life … another person … I can relate to.
… I do not use to talk much … the painting is for me a good help … makes me comfortable to start a conversation ….

**Mentally Present**

… when I talk about a painting … I communicate my feelings … a help to concentrate ….
… I feel present … much more present … in body and mind … I was aware of … awake ….
… I do not take for granted … experience attendance … presence and reality ….
… it is a way to capture time … be present ….
… a feeling of timelessness … time broadens … walk away from reality ….
… you are totally fascinated … time goes by truly fast … absorbed of the painting … the dialogue force or help you to concentrate ….

### 4.2. Part 2

**Based on Question 2**

Participants were asked to respond to the question; what meaning has music, books, movies, theaters, museums in your life? The results are presented as percentage of participants (*n* = 20) because participants replied to this question in one or two words. Therefore, the results are presented in percentage as to get a clear picture of the result.

78% of the participants answered:

Makes me happy, gives energy, gives inspiration.

22% of the participants answered:

Makes me harmonious, gives peace, open new windows.

### 4.3. Part 3

**Based on Question 3**

The meaning of gallery visits with structured dialogues compared to gallery visits without structured dialogues.

52% of the participants answered:

The dialogue increases inspiration, the dialogue makes you involved, you learn from the dialogue, the dialogue stimulates curiosity.

48% of the participants answered:

The dialogue starts thoughts, the dialogue makes you expectant, and the dialogue is thrilling.

### 4.4. Part 4

**Based on Question 4**

A questionnaire with four questions was used for reflections two months after the study was finished.

1. Which art form do you prefer?
2. How should this art form be developed in order to reach many people?
3. Why is the art form you prefer important for you?
4. The art form you prefer—has your preferences changed during the art dialogues?

**Which Art Form Do You Prefer?**

… many art forms are needed … depends on your state of mind … awareness … consciousness ….
… different art forms are needed … a resource … put into words … thinking … feelings ….
… sometimes I need a book … sometimes I need to listen to music … it depends on my frame of mind.

**How Should the Art Form You Prefer Be Developed in Order to Reach Many People?**

… art should be prioritized in society by the government.
… every hospital … paintings and sculptures that welcome the visitor ….
… hospital personnel … start art dialogues with patients ….
… different perspective … economically, politically, and culturally must be considered ….
… you have to be offered opportunities early in life … in order to appreciate … learn to express oneself … find your own way … it might be difficult … if you do not start early in life.
.… start gallery for amateurs … in nursing care ….

**Why is The Art Form You Prefer Important for You?**

… you learn new things about yourself … about the society ….
… a way to loosen up … a way to be alert and aware ….
*The Art Form You Prefer—Has Your Preferences Changed during the Art Dialogues?*
All respondents answered no to this question.

## 5. Discussion

The aim of the study was to investigate the value of adding structured art dialogues in small group setting.

The art dialogue starts with observation. Participants carefully observe the paintings to be able to choose one painting they prefer. Then they were asked to describe this painting according to form, colour, and representation. Then in the interpretation phase, the participants were asked to use imagination. In the reflection phase, they listen to and discuss with fellow participants.

It was considered important that the paintings the participants could choose among were in balance concerning complexity and level of representativeness with the onlooker's ability to perceive them [2, 4, 38, 39].

The main results are that the participants expressed positive value effects of an art dialogue structure. This can be seen in light of the communication process as a complex experience of personal experience [4]. In the present study this could be exemplified by an excerpt from a respondent: “I do not use to talk much … the painting is for me a good help … makes me remember … and it starts conversation ….” The respondents expressed that art is an important part of their life. It is expressed in words like makes me happy, gives energy, and gives inspiration. Similar findings could be found in a study about older adults who express the importance of art in their life. They speak of timelessness and spacelessness and a source of gratification [5]. In addition participants express that art dialogues increase and raise associations. It is expressed in words like “… the painting … shows the precision … of people… forgotten … long ago …” and “… the painting … makes me remember … I have never thought of … since I was four or five years of age.” Similar thinking is expressed in previous research [5, 12]. The structured art dialogue is also regarded as door-openers. Expressions from respondents in the present study show this aspect: ” … I like this way to get close to the painting … is a way to my inner life …” and “… it is easy to open a conversation … leads to further conversation … one subject leads to another subject ….” Similar findings are to be found in previous research [19]. A further result in the present study is that the respondents expressed “mentally presence” when engaged in the art dialogues. It is expressed in words like “… I feel present … much more present … in body and mind… I was aware of … awake …” and “… you are totally fascinated … time goes by truly fast … absorbed of the painting … the dialogue force or help you to concentrate ….” Similar thinking is expressed in several studies [10, 11, 40, 42, 43]. To share emotions and to discuss visual art play an important role for a successful gallery visit. Supporting the spectator could predict a new quality based on the content of the painting.

### 5.1. Weaknesses and Strength in the Method

To the best of the authors' knowledge, qualitative semistructured questionnaires were the best approach to answer the research question. However, limitations of the questionnaire could appear for various reasons. It was obvious that the respondents found it difficult to put into words their experiences of art dialogues. However, when the respondents were told that they cannot write anything that is wrong, they experience a release. A weakness in the present study could be that objective data was not collected. Nonetheless, the main focus of the study has been the participants' perception and points of view about adding structured dialogues to a gallery visit. Still the problem with accidental sampling is that available subjects might be atypical of the population with regard to the variables being measured.

A bias factor that could not be excluded is that the participants could feel forced to give positive opinions about the importance of art dialogues, because it was the artist who assisted participants in the art dialogues. However, participants were not supposed to write their names on the written responses. In addition, they placed their responses in an envelope that was sent to the researcher. Thus, the text could not be identified to a specific person.

## 6. Conclusion

Participants experienced the visual art dialogue program as relevant and fruitful. The present study supported the view that meaningful art stimulation, related to a person's experiences and expectations, will be of importance in peoples' life.

It is to be hoped that the art dialogues' program could be implemented in hospital settings as a fruitful working tool for health care professionals. It might contribute to complementary ways of communication with patients. Art should be regarded as a source of inspiration. It builds upon a person's knowledge and personal experiences. It offers a cognitive and emotional tool in the communication process.

We have to take into consideration the importance a visual art dialogue can have for health professionals and patients. The approach in this study demonstrates one way of using visual art as a means of communication. However, more data are needed in order to explore the means by which art communication can be successful.
